# Anatomical network modules of the human central nervous-craniofacial skeleton system

**DOI:** 10.3389/fneur.2023.1164283

**Published:** 2023-08-02

**Authors:** Gele Qing, Fucang Jia, Jianwei Liu, Xiling Jiang

**Affiliations:** ^1^Affiliated Hospital of Chifeng University, Chifeng, China; ^2^Shenzhen Institute of Advanced Technology, Chinese Academy of Sciences, Shenzhen, China

**Keywords:** anatomical network analysis, module, central nervous, craniofacial skeleton, craniofacial development

## Abstract

Anatomical network analysis (AnNA) is a systems biological framework based on network theory that enables anatomical structural analysis by incorporating modularity to model structural complexity. The human brain and facial structures exhibit close structural and functional relationships, suggestive of a co-evolved anatomical network. The present study aimed to analyze the human head as a modular entity that comprises the central nervous system, including the brain, spinal cord, and craniofacial skeleton. An AnNA model was built using 39 anatomical nodes from the brain, spinal cord, and craniofacial skeleton. The linkages were identified using peripheral nerve supply and direct contact between structures. The Spinglass algorithm in the igraph software was applied to construct a network and identify the modules of the central nervous system-craniofacial skeleton anatomical network. Two modules were identified. These comprised an anterior module, which included the forebrain, anterior cranial base, and upper-middle face, and a posterior module, which included the midbrain, hindbrain, mandible, and posterior cranium. These findings may reflect the genetic and signaling networks that drive the mosaic central nervous system and craniofacial development and offer important systems biology perspectives for developmental disorders of craniofacial structures.

## Introduction

Anatomical network analysis (AnNA) is a tool for understanding the mosaic relationships of anatomical structures using network engineering theory for the quantitative analysis of anatomical structures (1). This method deconstructs complex anatomical systems as a network model that comprises individual anatomical elements or structures as nodes, and their relationships as linkages or network edges, to model complex topology, and derive an understanding of the underlying developmental biology (2). Essentially, an anatomical network module is a group of anatomical elements that are more densely connected than these are with others outside the module. The human head is a highly complex anatomical structure. The AnNA approach has modeled the adult human head which consists of 181 anatomical units into 10 musculoskeletal modules (3). The human brain has also been modeled using AnNA, in which the brain sulci and its imprints on the endocranium were used to define anatomical elements, providing 15 regions that can be modeled into anterior and posterior blocks.

Molecular biology, evolutionary developmental biology, and clinical medicine have all indicated that the human head is an organic integrity of the brain-skull-face, nerve-muscle-skeleton composite (4–7). The embryonic developmental processes of the brain and face are closely correlated due to a common embryonic origin and adjacent anatomical location. An obvious corollary is the frequent association of craniofacial syndromes with the central nervous system or sensory dysfunction (8–10). Furthermore, neurobehavioral syndromes caused by central nervous system alterations, such as schizophrenia or autism, are associated with increased incidences of craniofacial malformations (11), suggesting that the brain and face are extensively and closely correlated in both physiological and pathological states. Thus, the central nervous system and craniofacial structures appear as mutually interacting and influencing entireties. Although the brain and craniofacial structures have been separately analyzed as anatomical networks (12–16), these have not been analyzed as a single composite. From the evolutionary perspective of vertebrate food acquisition, several studies (17–21) have revealed that the transition to mobile hunting necessitated the development of a complex sensory system alongside craniofacial skeletal modification. Therefore, the present study aimed to analyze the anatomical network comprising the central nervous and craniofacial skeleton systems as a whole.

## Materials and methods

### Nodes and links

The nodes and links of the craniofacial skeleton were defined, as previously described (15). Consistent with the anatomic terms for embryonic development, the central nervous system was divided into the following sections: left forebrain (develops into the left telencephalon and left diencephalon), right forebrain (develops into the right telencephalon and right diencephalon), left midbrain, right midbrain, left hindbrain (develops into the left pons, left cerebellum, and left medulla oblongata), right hindbrain (develops into the right pons, right cerebellum, and right medulla oblongata), left spinal cord, and right spinal cord. The left and right parts of the brain and spinal cord connect at the central axis using direct contact or nerve junctions. Therefore, the central axis of the brain was defined based on the anatomical position. The other sections of the brain and spinal cord also connect through direct contact or nerve junctions.

The linkages or connections between the craniofacial skeleton and the central nervous system were defined based on two linkages. The first linkage type was a connection via the peripheral nerve supply. For example, the nociceptor locations on the periosteum of the mandible indicate that the mandible is the initial node, the trigeminal nerve that supplies these is the link, and the hindbrain houses the trigeminal nuclei form the terminal node. The second linkage type was through direct contact. These two types of linkages often coexist. For example, the parietal region is connected to the hindbrain through the trigeminal nerve, while the meninges lie between the brain and skull, allowing the parietal region to be directly connected to the forebrain through the meninges (22, 23). For special sensory organs of the head, including the eyes, nose, and ears, similar definitions were followed, except for some minor differences for the eyes. Since an eye is surrounded by seven periorbital bones (frontal, sphenoid, ethmoidal, palatine, lacrimal, maxilla, and zygomatic), these are all connected to the forebrain through the optic nerve, and the hindbrain through the trigeminal nerve [SG; (24)].

The present study established a network model, which included 39 nodes (nine brains, three spinal cords, and 27 craniofacial skeleton nodes). Codes were assigned for each pair of elements. A value of 0 was entered when the elements were not linked, and a value of 1 was entered when the elements were linked. The adjacency matrix is presented in Table 1.

**Table 1:** The adjacency matrix*.

	1	2	3	4	5	6	7	8	9	10	11	12	13	14	15	16	17	18	19	20	21	22	23	24	25	26	27	28	29	30	31	32	33	34	35	36	37	38	39
1		**1**		**1**			**1**			**1**	**1**		**1**		**1**	**1**	**1**	**1**	**1**			**1**	**1**	**1**	**1**	**1**	**1**												
2	**1**		**1**		**1**																																		
3		**1**				**1**			**1**	**1**		**1**		**1**	**1**	**1**	**1**	**1**	**1**			**1**	**1**	**1**	**1**	**1**	**1**												
4	**1**				**1**		**1**																																
5		**1**		**1**		**1**		**1**																															
6			**1**		**1**				**1**																														
7	**1**			**1**				**1**		**1**	**1**		**1**		**1**	**1**		**1**	**1**	**1**		**1**		**1**		**1**		**1**					**1**		**1**	**1**			
8					**1**		**1**		**1**																												**1**		
9			**1**			**1**		**1**		**1**		**1**		**1**	**1**		**1**	**1**	**1**		**1**		**1**		**1**		**1**	**1**						**1**	**1**			**1**	
10	**1**		**1**				**1**		**1**		**1**	**1**	**1**	**1**	**1**																					**1**		**1**	
11	**1**						**1**			**1**		**1**	**1**		**1**			**1**																					
12			**1**						**1**	**1**	**1**			**1**	**1**			**1**																					
13	**1**						**1**			**1**	**1**				**1**	**1**													**1**						**1**	**1**			
14			**1**						**1**	**1**		**1**			**1**		**1**													**1**					**1**			**1**	
15	**1**		**1**				**1**		**1**	**1**	**1**	**1**	**1**	**1**		**1**	**1**	**1**	**1**							**1**	**1**	**1**											
16	**1**		**1**				**1**						**1**		**1**			**1**				**1**																	
17	**1**		**1**						**1**					**1**	**1**			**1**					**1**																
18	**1**		**1**				**1**		**1**		**1**	**1**			**1**	**1**	**1**		**1**	**1**	**1**	**1**	**1**	**1**	**1**														
19	**1**		**1**				**1**		**1**						**1**			**1**		**1**	**1**	**1**	**1**	**1**	**1**	**1**	**1**	**1**											
20							**1**											**1**	**1**		**1**	**1**																	
21									**1**									**1**	**1**	**1**			**1**																
22	**1**		**1**				**1**									**1**		**1**	**1**	**1**			**1**	**1**		**1**		**1**											
23	**1**		**1**						**1**								**1**	**1**	**1**		**1**	**1**			**1**		**1**	**1**											
24	**1**		**1**				**1**											**1**	**1**			**1**																	
25	**1**		**1**						**1**									**1**	**1**				**1**																
26	**1**		**1**				**1**								**1**				**1**			**1**					**1**	**1**											
27	**1**		**1**						**1**						**1**				**1**				**1**			**1**		**1**											
28							**1**		**1**						**1**				**1**			**1**	**1**			**1**	**1**												
29													**1**																		**1**								
30														**1**																		**1**							
31																													**1**				**1**						
32																														**1**				**1**					
33							**1**																								**1**								
34									**1**																							**1**							
35							**1**		**1**				**1**	**1**																									
36							**1**			**1**			**1**																								**1**		**1**
37								**1**																												**1**		**1**	
38									**1**	**1**				**1**																							**1**		**1**
39																																				**1**		**1**	

^*^Matrix element labels: (1) left forebrain (left telencephalon and left diencephalon), (2) forebrain central axis, (3) right forebrain (right telencephalon and right diencephalon), (4) left midbrain (left midbrain), (5) midbrain central axis, (6) right midbrain (right midbrain), (7) left hindbrain (left pons, left cerebellum, and left medulla oblongata), (8) hindbrain central axis, (9) right hindbrain (right pons, right cerebellum, and right medulla oblongata), (10) occipital, (11) parietal.left, (12) parietal.right, (13) temporal.left, (14) temporal.right, (15) sphenoid, (16) zygomatic.left, (17) zygomatic.right, (18) frontal, (19) ethmoidal, (20) nasal.left, (21) nasal.right, (22) maxilla.left, (23) maxilla.right, (24) lacrimal.left, (25) lacrimal.right, (26) palatine.left, (27) palatine.right, (28) vomer, (29) malleus.left, (30) malleus.right, (31) incus.left, (32) incus.right, (33) stapes.left, (34) stapes.right, (35) mandible, (36) left spinal cord, (37) spinal cord central axis, (38) right spinal cord, and (39) hyoid.bone. A value of 1 refers to a linkage or edge, while a value of 0 refers to no linkage.

### Network construction using the Spinglass algorithm

The Spinglass algorithm, which is a community detection algorithm that enables modularity recognition in networks, was applied for the network construction (25, 26). Pairwise interactions in a spin glass-based system were modeled on the premise that the network edges connect the nodes in similar ‘spin states,’ and that these represent the biological contexts in this case. Previous research has demonstrated the stable and superior performance of Spinglass in smaller networks to achieve good community partition (25, 26). The present study utilized the Spinglass function in igraph (27) to construct the network: the number of spins = 2, and the other parameters were set as default for Spinglass.

## Results

**Figure 1:**
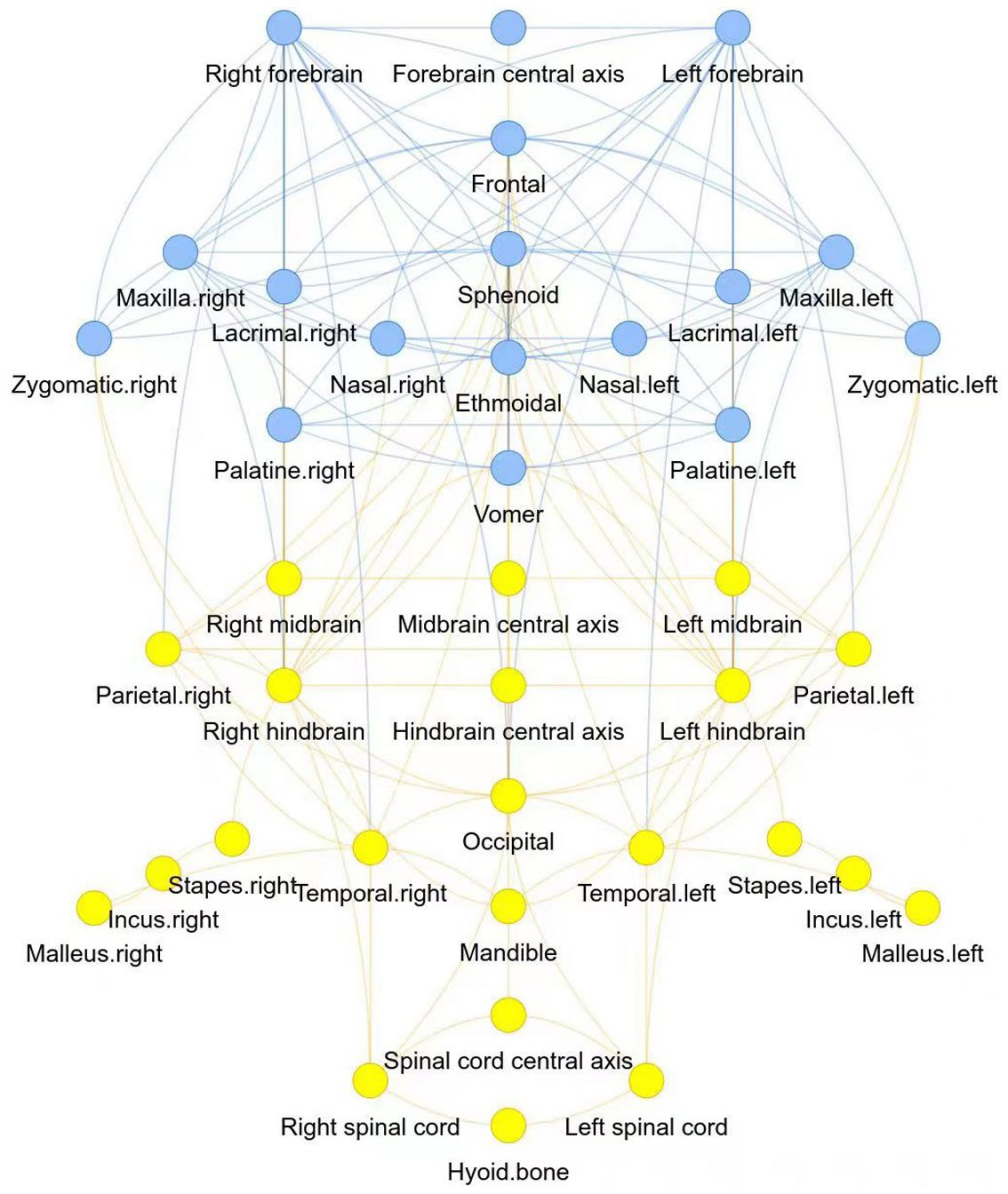
Two modules of the human central nervous-craniofacial skeleton were identified using the AnNA approach: module 1 was presented in blue, and module 2 was presented in yellow.

Two modules were identified using the AnNA approach (Table 2). Module 1 comprised of the left and right forebrain, along with the forebrain central axis, as central nervous system structures, and the mid and upper face complex, including the sphenoid, zygomatic, frontal, ethmoidal, nasal, maxilla, lacrimal, palatine and vomer bones. The posterior module included the midbrain and hindbrain structures, and spinal cord, along with the parietal, temporal, and mandibular regions, and the bones of the ear (Figure 1).

**Table 2:** Modules of the human central nervous-craniofacial skeleton system identified using spinglass algorithm.

Modules	Central nervous system structures	Craniofacial structures
Module 1	Left forebrain (left telencephalon and left diencephalon), forebrain central axis, and right forebrain (right telencephalon and right diencephalon)	Sphenoid, Zygomatic.left, Zygomatic.right, Frontal, Ethmoidal, Nasal.left, Nasal.right, Maxilla.left, Maxilla.right, Lacrimal.left, Lacrimal.right, Palatine.left, Palatine.right, and Vomer
Module 2	Left midbrain, midbrain central axis, right midbrain, left hindbrain (left pons, left cerebellum, and left medulla oblongata), hindbrain central axis, right hindbrain (right pons, right cerebellum, and right medulla oblongata), left spinal cord, spinal cord central axis, and right spinal cord	Occipital, Parietal.left, Parietal.right, Temporal.left, Temporal.right, Malleus.left, Malleus.right, Incus.left, Incus.right, Stapes.left, Stapes.right, Mandible, and Hyoid.bone

Furthermore, we employed two algorithms, namely the community_leading_eigenvector algorithm and an improved version of the Spinglass algorithm, to recompute the modularization results of the 39 selected nodes. These results were compared with the modularization outcomes obtained using the original Spinglass algorithm. The modularization results obtained from the community_leading_eigenvector algorithm were largely consistent with those obtained from the Spinglass algorithm (Table 3), except for the assignment of the sphenoid bone module. However, this discrepancy is not unexpected given the sphenoid bone’s position at the interface of two modules, being influenced by both during development and closely associated with both.

**Table 3:** Modules of the human central nervous-craniofacial skeleton system identified using the community-leading eigenvector algorithm.

Modules	Central nervous-craniofacial skeleton structures
Module 1	Left forebrain, Forebrain central axis, Right forebrain, zygomatic. Left, zygomatic. Right, frontal, ethmoidal, nasal.left, nasal.right, maxilla.left, maxilla.right, lacrimal.left, lacrimal.right, palatine.left, palatine.right, vomer
Module 2	Left midbrain, Midbrain central axis, Right midbrain, Left hindbrain, Hindbrain central axis, Right hindbrain, occipital, parietal. Left, parietal.right, temporal.left, temporal.right, sphenoid, malleus.left, malleus.right, incus.left, incus.right, stapes.left, stapes. Right, mandible, Left spinal cord, spinal cord central axis, Right spinal cord, hyoid. Bone

In anatomical networks, asymmetry in modularization results is a common challenge encountered with the Spinglass algorithm. To address this, we made the following modifications to the algorithm: (1) we initiated the updating of spin states with nodes located on the anatomical network’s symmetry axis; (2) during the update of a node’s spin state, we checked if it resided on the axis. If it did, only that node was updated. Otherwise, both the node and its symmetrical counterpart had their spin states updated to ensure consistency among symmetrical nodes. By implementing these modifications, each iteration of the Spinglass algorithm produced symmetric results, where nodes within each module were arranged symmetrically. The computed results obtained using the modified Spinglass algorithm align with the original Spinglass algorithm results (Table 4).

**Table 4:** Modules of the human central nervous-craniofacial skeleton system identified using a modified spinglass algorithm.

Modules	Central nervous-craniofacial skeleton structures
Module 1	‘Lacrimal left,’ ‘Lacrimal right,’ ‘Palatine left,’ ‘Palatine right,’ ‘Nasal left,’ ‘Nasal right,’ ‘Maxilla left,’ ‘Maxilla right,’ ‘Left forebrain,’ ‘Right forebrain,’ ‘Vomer,’ ‘Forebrain central axis,’ ‘Sphenoid,’ ‘Frontal,’ ‘Ethmoidal,’ ‘Zygomatic left,’ ‘Zygomatic right.’
Module 2	‘Malleus left,’ ‘Malleus right,’ ‘Mandible,’ ‘Parietal left,’ ‘Parietal right,’ ‘Occipital,’ ‘Left hindbrain,’ ‘Left midbrain,’ ‘Left spinal cord,’ ‘Temporal left,’ ‘Temporal right,’ ‘Midbrain central axis,’ ‘Right hindbrain,’ ‘Right midbrain,’ ‘Right spinal cord,’ ‘Hindbrain central axis,’ ‘Incus left,’ ‘Incus right,’ ‘Spinal cord central axis,’ ‘Stapes left,’ ‘Stapes right,’ ‘Hyoid bone.’

## Discussion

The present study established a central nervous system-craniofacial skeleton anatomical network model. The overall findings were consistent with the cell biological, molecular, and genetic observations, and the collective evidence revealed that the brain and craniofacial structures are parts of a closely co-evolved system (6, 7, 11). The facial primordium originates from the cranial neural crest that arises in the dorsal most aspect of the forming neural tube. The neural tube gives rise to the brain and spinal cord, which explains the shared histoembryological homology of the brain and craniofacial regions. In addition, the signal centers in the frontonasal ectodermal zone of the brain regulate the growth and development of the face, while the signaling molecules from the face are involved in controlling forebrain growth. Thus, a “brain and face dialogue” occurs via signaling channels. The brain also serves as a structural platform for determining the location of the facial primordium. Brain growth patterns and speed directly impacts facial morphogenesis as a synchronous process. Several anatomical studies have used network analysis to model the development, function, and evolution of various morphological systems (2). Previous AnNA studies of the human head have analyzed the craniofacial musculoskeletal system (2, 3, 15, 28)alone, but these did not consider the brain-skull-face as organic integrity. During the initial phase of our study, we carefully defined 39 nodes based on the comprehensive work of Borja Esteve-Altava et al. and Vance Powell et al. (3, 15), which provided detailed documentation of skeletal, cartilaginous, and muscular components in 12 cadaveric specimens. These nodes were thoughtfully selected to represent specific anatomical structures and their corresponding connections or attachments on the normal adult human head. In addition, the peripheral nerve supply was considered as a link between the nodes of the craniofacial and central nervous system nodes, enabling a systems view of these structures based on embryonic development and adult anatomy.

In addition, the anatomical network model of the craniofacial skeleton included pairs of the left and right nodes, which are symmetrically distributed along the central nervous system Furthermore, the anteroposterior (A-P) axis is consistent with the embryological, molecular patterning of the human head. Biological evidence has demonstrated that the development of the face and other non-axial bilaterally symmetric structures occurs along the A-P axis (11). The morphogenesis at sites of non-axial induction, which includes the face, is dependent on the orchestration of neural crest-derived mesenchymal-epithelial interactions (29–31). These coordinated developmental processes allow for the integration of sensory and motor functions for the execution of essential behaviors. The symmetrical distribution of network nodes along the central axis was consistent with the symmetrical development of neural crest-derived peripheral nerves, neural circuits, and peripheral structures. The anterior module in the network consisted of the forebrain, anterior cranial base, and upper-middle face, and the posterior module consisted of the midbrain, hindbrain, mandible, and posterior cranium. The lateral edge of the forebrain produces neural crest cells that migrate to the frontonasal mass, while neural crest cells that emigrate from both the midbrain and hindbrain migrate to the first branchial arch (32), and distinct cell populations have been noted. The early neural crest comprises Hox^−^cells that correspond to the anterior cranial neural crest, and Hoxb2^+^ cells that contribute to the development of the mandible (31). Signaling involved in the forebrain and upper jaw development arises from the frontonasal ectodermal zone, to regulate the upper jaw development and facial regulation of forebrain growth (33). In summary, the structures of the forebrain, anterior cranial base, and upper-middle facial midline are co-regulated, leading to a highly coordinated anatomical and functional relationship (8). The skull vault, cranial base, and meninges are all derived from two sources: the frontal regions are neural crest-derived, and the parietal regions have a mesodermal origin (34). The meninges that envelop the forebrain are derived from the neural crest, while the meninges that envelop the midbrain and hindbrain are derived from the cephalic mesoderm. The neural crest-derived portions of the meninges have evolved in tandem with the rostral parts of the brain and skull, suggesting commonalities in development in response to shared signals (33). In addition, the anterior cranial base is solely derived from the neural crest, and the posterior cranial base originated from the paraxial mesoderm. Since the anterior cranial base has had a longer growth phase, it has exerted a greater influence on facial growth. This was also directly connected to the upper-middle face, forming the ethmomaxillary complex (35).

In the current study, the identification of two anatomical network modules along the A-P axis is consistent with the spatial arrangement observed during the embryonic development of FGFs, Wnts, and BMPs signaling molecules. FGFs are known to regulate the development of anterior anatomical structures through gene networks, while Wnts and BMPs signaling molecules are involved in the development of posterior anatomical structures through gene networks. Moreover, these signaling molecules establish boundaries and regulatory domains through interactions of cross-inhibition and cross-self-regulation. Hence, the boundaries of the identified anatomical network modules may correspond to the boundaries defined by FGFs, Wnts, and BMPs signaling molecules during embryonic development (36, 37), although further confirmation is necessary to validate this correlation.

In the AnNA reported in the present study, the physical connection between the sensory nervous system and the craniofacial skeleton was consistent with the relationship between the neural crest and ectodermal placodes, which is evident during cranial sensory development. Notably, the sensory nervous system has a close connection to the craniofacial skeleton. Sensory placodes and neural crest cells are among the key cell populations that have facilitated the evolution of vertebrates. Shared molecular mechanisms underlie these processes before the establishment of definitive lineages, and anterior–posterior patterning forms the basis of sensory placode identity (20). The AnNA modules identified in the present study correspond to such anterior and posterior patterning of the placodes. Bones that are associated with sight (lens placode) and smell (olfactory placode) were included in the anterior AnNA module, while those associated with auditory sense (otic placode) were included in the posterior AnNA module, which included the bones of the ear.

Some inconsistencies between the results and other studies were evident. In the present study, the zygomatic arch was included in the anterior cranial module, while other studies reported that this was included in the posterior cranial module (20). This could be attributed to the overlapping nature of the anterior and posterior modules or its role as a bridging structure between the anterior and posterior skull. Since different algorithms may lead to different conclusions, there is a need to compare and identify the most stable community network detection algorithm. In summary, an AnNA network model was constructed, and this appeared to represent the genetic and signaling networks underlying the coordinated development of the craniofacial skeleton and the brain. These findings reiterate AnNA is an important tool for studying vertebrate evolution, embryonic development, and disease correlation.

## Conclusion

Our study utilized anatomical network analysis (AnNA) to investigate the modular organization of the human head, including the central nervous system and craniofacial skeleton. We identified significant structural and functional correlations between the human brain and facial structures, implying the presence of a co-evolved anatomical network. Incorporating 39 anatomical nodes from the brain, spinal cord, and craniofacial skeleton, our comprehensive AnNA model encompassed peripheral nerve supply and direct structural connections. By applying the Spinglass algorithm, we successfully identified two distinct modules within the anatomical network of the central nervous system and craniofacial skeleton. These findings offer valuable insights into the genetic and signaling networks that govern the intricate development of these interconnected systems.

## Data availability statement

The original contributions presented in the study are included in the article/supplementary material, further inquiries can be directed to the corresponding author.

## Author contributions

GQ: investigation, conducting a research and investigation process, specifically performing the experiments, data and evidence collection, methodology, development or design of methodology, and creation of models. FJ: formal analysis, application of statistical, mathematical, computational, other formal techniques to analyze or synthesize study data, software programming, software development, designing computer programs, implementation of the computer code and supporting algorithms, and testing of existing code components. JL: data curation, management activities to annotate (produce metadata), scrub data, and maintain research data (including software code, where it is necessary for interpreting the data itself) for initial use and later re-use, resources, provision of study materials, reagents, materials, patients, laboratory samples, animals, instrumentation, computing resources, or other analysis tools. XJ: conceptualization, ideas, formulation or evolution of overarching research goals and aims, funding acquisition, acquisition of the financial support for the project leading to this publication, project administration, management and coordination responsibility for the research activity planning and execution. All authors read and approved the final manuscript.

## Funding

The study was financially supported by grants from the National Natural Science Foundation of China (No. 81960208), the Key Projects of Inner Mongolia (No. 2022YFSH0046), and the Inner Mongolia International Science and Technology Cooperation Project (Three-dimensional image analysis of craniomaxillofacial deformity based on the neural crest anatomic network module).

## Conflict of interest

The authors declare that the research was conducted in the absence of any commercial or financial relationships that could be construed as a potential conflict of interest.

## Publisher’s note

All claims expressed in this article are solely those of the authors and do not necessarily represent those of their affiliated organizations, or those of the publisher, the editors and the reviewers. Any product that may be evaluated in this article, or claim that may be made by its manufacturer, is not guaranteed or endorsed by the publisher.
